# Effect of Heparin Administration during Coronary Angiography on Vascular or Peripheral Complications: A Single-Blind Randomized Controlled Clinical Trial

**Published:** 2013-12

**Authors:** Mohsen Gharakhani, Farzad Emami

**Affiliations:** Department of Cardiology, School of Medicine, Hamadan University of Medical Sciences, Hamadan, Iran

**Keywords:** Coronary angiography, Heparin, Hemorrhage, Iran

## Abstract

**Background: **Coronary angiography consists of the selective injection of contrast agents in coronary arteries. Optimal strategy for heparin administration during coronary angiography has yet to be determined. We assessed the effect of heparin administration during coronary angiography on vascular, hemorrhagic, and ischemic complications.

**Methods:** Five hundred candiates for diagnostic coronary angiography (femoral approach) were randomly divided into case (intravenous Heparin [2000-3000 units]) and control (placebo) groups. Assessment included vascular complications like groin hematoma, retroperitoneal hematoma, pseudoaneurysm, active hemorrhage, cerebral ischemia, and clot formation in the catheter or the sheath during angiography. Information was obtained about the patients’ age, sex, and hypertension and diabetes mellitus history. Patients with severe peripheral vascular disease, aortic stenosis, history of coagulopathy, and angiography over 30 minutes were excluded.

**Results:** Nine patients from each group were excluded. The remaining 482 patients included 285 (59.1%) men and 197 (40.9%) women. In the case group (n=241), 7 (2.9%) patients experienced active hemorrhage at the site of angiographic puncture, 2 (0.83%) developed groin hematoma, and 8 (3.32%) experienced clot formation during angiography, while the corresponding figures for the control group (n=241) were 3 (1.24%), 2 (083%), and 13 (5.39%), respectively. No significant differences were found in hemorrhagic, ischemic, and vascular complications between the two groups.

**Conclusion: **Heparin administration during coronary angiography had no effect on clot formation as well as hemorrhagic, ischemic, and vascular complications in our patients.

**Trial Registration Number: **IRCT201202199080N1

## Introduction

Coronary artery disease (CAD) is the major culprit for mortality in industrial countries, with various risk factors having been identified for this disease. A reduction in the number of patients suffering from CAD requires the early identification of these risk factors. Old age, male sex, and familial history of early CAD are deemed major non-modifiable CAD risk factors,^1^ whereas systemic arterial hypertension, hyperlipidemia, metabolic syndrome, insulin resistance, diabetes mellitus, and smoking are among the modifiable risk factors for CAD. Other risk factors include obesity, low physical activity, hyperhomocysteinemia, high lipoprotein (a) or fibrinogen levels, mental stress, depression, and other novel risk factors such as high-sensitive C**-**reactive protein (CRP) levels.^2^

Coronary angiography is a relatively safe diagnostic procedure insofar as its rates of major complications, i.e. death, stroke, and myocardial infarction, stand at less than 0.1%.^3^ This modality is still regarded as the gold standard for identifying stenosis caused by atherosclerosis and, in addition, yields reliable results for deciding whether to continue drug therapy or to use invasive methods for treatment. 

As an anticoagulant, heparin prevents thrombosis and inhibits natural homeostasis by creating a complex with anti-thrombin III and enhancing its effect. It potentially increases the possibility of vascular and hemorrhagic complications such as hematoma at the site of catheterization after initial hemostasis, retroperitoneal hemorrhage, and pseudoaneurysm at the site of femoral artery puncture, all of which might necessitate diagnosis and management.^4^^,^^5^ Consequently, when we use anticoagulant therapy, the risk of bleeding during the procedure must be balanced against the risk of thrombotic event. 

Be that as it may, heparin use has some limitations not only due to its unpredictable effects^6^^-^^10^ but also due to its prothrombotic properties owing to platelet activation, poor control on von Willebrand factor release, and rebounding of thrombin generation when it is discontinued.^11^^,^^12^ Use of anticoagulant medication during elective and primary percutaneous coronary intervention has been generally supported by previous research.^13^ Researchers have long been evaluating the incidence of the hemorrhagic complications of heparin in coronary angiography as opposed to its protective effects on reducing ischemic coronary events during and after angiography. We aimed to assess the advantages and disadvantages of heparin administration during coronary angiography with respect to clot formation as well as vascular, ischemic, and hemorrhagic complications.

## Patients and Methods

**Figure 1:**
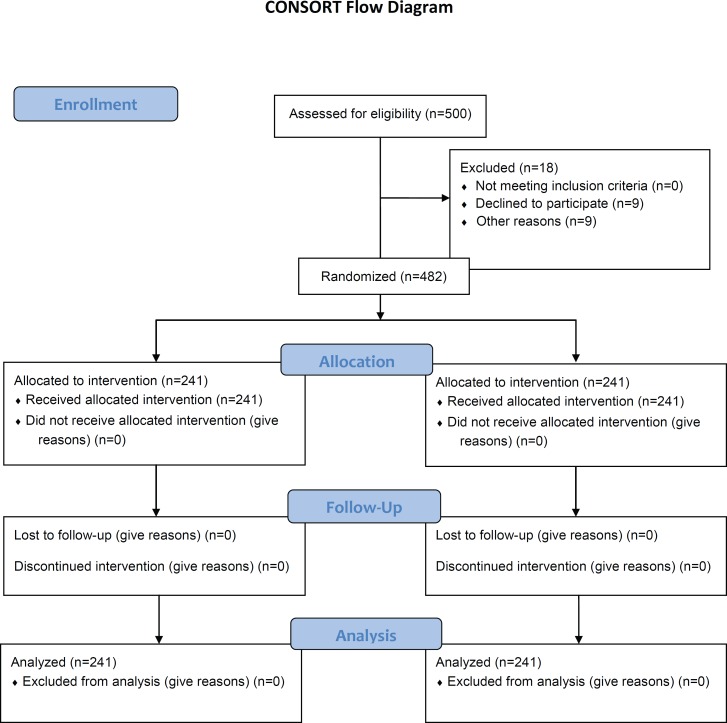
This flow diagram depicts the progress through the phases of this parallel randomized trial of the two study groups.

This single-blind, randomized controlled trial was conducted in Ekbatan Hospital, in the western Iranian city of Hamadan, between 2007 and 2008. The trial was approved by the local Human Subject Review Board of Hamadan University of Medical Sciences (No: 4226) and indexed by the Iranian Register of Clinical Trials (No: 201202199080N1). The patients all volunteered to enroll in the study and signed written informed consent (figure 1).

We enrolled all patients with CAD who were referred to Ekbatan Hospital for coronary angiography with the following criteria: (1) typical chest pain; (2) positive exercise test; (3) regional wall motion abnormality in echocardiography; (4) positive gated technetium 99m sestamibi single emission computed tomography (Tc99m-MIBI-SPECT); (5) previous history of Coronary Care Unit (CCU) admission due to acute coronary syndrome; and (6) history of myocardial infarction. Patients with the following criteria were excluded from the study: (1) severe aortic stenosis; (2) severe peripheral vascular disease; (3) history of coagulopathy; and (4) duration of angiographic procedure more than 30 minutes.

Based on statistical formulae and considering a 10% probability of dropout, a sample of 500 patients was estimated for this study. The patients were randomized into two equal intervention (receiving heparin) and control (receiving placebo) groups using a systematic method so that the first patient was randomly assigned to one of the two groups through coin tossing and then the subsequent patients were assigned to either group one at a time. The study was conducted using a single-blind design, and while the researchers knew which patients had been assigned to the intervention or control groups, the patients were not aware of the administered intervention. The first group received 2000-3000 units intravenous heparin based on the body weight (less than 60 kg received 2000 units; 60-80 kg received 2500 units; and more than 80 kg received 3000 units) during coronary angiography, and the control group received normal saline as placebo.

The outcomes of interest were periangiographic complications, including active bleeding, groin hematoma, and clotting. Active bleeding was defined as bleeding from the puncture site of longer than 15 minutes after manual compress, groin hematoma was defined as a painful and swollen area with bruise ≥5 cm, and clotting was defined as the presence of clot at the site of the femoral access and sheath. 

Coronary angiography was performed through six Fr sheaths via the femoral artery, using a modified Seldinger technique. Visipaque was used as contrast medium. The femoral arterial sheath was removed immediately after the procedure and was compressed manually for a minimum of 15 minutes until homeostasis occurred. Mobilization was permitted for a minimum of 8 hours after the sheath removal. The angiography puncture sites were assessed at 4 and 24 hours after the completion of the procedure. 

The Pearson Chi-square test was employed for analysis at a significance level of 0.05 using statistical software Stata 11 (StataCorp, College Station, TX, USA).

## Results

Of the initial 500 patients, 9 were excluded from each group because of abnormal PT and PTT results. Of the remaining 482 patients, 285 (59.1%) patients were men and 197 (40.9%) were women, with an age range of 45-75 years. 

Four hundred twenty-five (88.2%) patients had ejection fraction >40%, while 57 (11.8%) had ejection fraction ≤40%. One hundred eighty-three (38%) patients had a history of hypertension, and 124 (25.7%) had a history of diabetes mellitus. Retroperitoneal hematoma and pseudoaneurysm at the site of the femoral puncture did not occur in any of the patients.

Hemorrhage occurred at the site of catheterization after initial hemostasis in 7 (2.9%) patients in the case and in 3 (1.24%) patients in the control group. Groin hematoma occurred in 2 (0.8%) patients in the case group and 2 (0.8%) patients in the control group. Clot formation in the catheter or sheath occurred in 8 (3.32%) patients of the case group and 13 (5.39 %) patients of the control group (table 1). 

**Table 1:** Frequency (%) of active hemorrhage at the site of catheterization after initial hemostasis, groin hematoma, and clotting in patients undergoing coronary angiography

**Complications**		**Case (n=241)**		**Control (n=241)**		**Total (n=482)**		**Chi-squared**	
		**Frequency**	**Per cent**	**Frequency**	**Per cent**	**Frequency**	**Per cent**	**P value**	**χ** ^2^
Active hemorrhage	Yes	7	2.90	3	1.24	10	2.08	0.201	1.6339
	No	234	97.10	238	98.76	472	97.92		
Groin hematoma	Yes	2	0.83	2	0.83	4	0.83	1.000	0.0000
	No	239	99.17	239	99.17	478	99.17		
Clotting	Yes	8	3.32	13	5.39	21	4.36	0.265	1.2447
	No	233	96.68	228	94.61	461	95.64		

No chest pain or new ECG change was observed in either of the groups, and nor were there any cerebral or peripheral vascular events in the patients of both groups.

## Discussion

All the studies that have hitherto sought to assess the efficacy of heparin administration during coronary angiography contradict each other in various aspects. 

The first aspect is the heterogeneity of the population. Some studies, including the present one, inherently focus on low-risk patients, which would lean toward a weaker conclusion in favor of heparin administration. Heterogeneity of the population segments the studies across different parameters, including age, sex, stage of the coronary disease along with underlying diseases like diabetes mellitus and hypertension, and other risk factors. The current study used wide exclusion criteria; this would inherently result in fewer occurrences of complicated cases. The male-to-female ratio in our study was around 6:4, and 25.7% of the patients had diabetes. Occurrence of local complications was low: only 2% of the patients in the case and control groups (1.2% hemorrhage and 0.8% groin hematoma, with no significant difference between the two groups). Local complication rates increase due to several factors such as age, obesity, hypertension, multiple punctures, and short duration of pressure to achieve homeostasis. Hypertension accounted for a large portion of our patient population (38%). However, most of our patients had acceptable condition. Indeed, 88.2% of the patients had ejection fraction >40%, which played an important role in the low incidence of complications. This should also be mentioned that operation time was less than 30 minutes. 

The second aspect is the difference in the arterial access, i.e. via femoral, brachial, or radial routes. It is a generally accepted practice to administer heparin via brachial and radial routes. Accordingly, studies with mixed arterial access approaches are more biased toward the positive efficacy of heparin. At present, 80% of coronary angiography cases are performed through the femoral route and 20% are done through the radial or brachial route.^14^ All the procedures in this study were done via the right femoral route. Wang Yq et al.^15^ were the first to report successful coronary angiography without the administration of heparin.

The third aspect is the difference in the administration route of heparin: intravenous or through the arterial sheath and in some studies even subcutaneous administration. We did not find any substantial evidence or prior studies in favor of either approach, but it could be hypothesized that administration through the arterial route is more effective due to locality advantages. 

The fourth aspect is the period of follow-up studies. Studies with longer periods for follow-up are more likely to find more accurate results in terms of complication rates. We assessed patients for a maximum of 24 hours after the procedure: this might have undervalued the occurrence of complications.

Based on the obtained results, we found no significant difference between the two groups with respect to hemorrhagic, ischemic, and vascular events or clot formation during coronary angiography. Furthermore, there was no statistical evidence that the prophylactic administration of heparin would increase serious groin bleeding or less atheroembolic complications. We did not observe any clinically significant thromboembolic events in our patients, and nor did we, in either of the two groups, observe any cases of chest pain, new ECG changes, or cerebral, peripheral, and vascular events. These findings might be due to the fact that the majority of the patients in this study were low-risk cases. (Among our study population, 88.2% had ejection fraction >40%.)

Our findings are consistent with those of another prospective study on 325 patients who had undergone coronary angiography. The researchers aimed to find the optimal strategy for administering heparin during coronary angiography; however, they did not find a significant difference between the two case (receiving heparin) and control groups with respect to ischemic, hemorrhagic, and vascular complications.^5^

Zibaeenezhad et al.^13^ reported no significant increase in ischemic complications after omission of heparin infusion in patients undergoing coronary intervention. Nevertheless, they reported that heparin would increase the occurrence of bleeding and vascular injury. Datta et al.^16^ reported no periprocedural ischemic complications during coronary angiography, which was performed without heparin, and they emphasized that coronary angiography through the femoral artery could be performed without heparin. A meta-analysis conducted by Johanne Silvain et al.^14^ reported that during percutaneous coronary intervention, Enoxaparin seemed to be superior to unfractionated heparin in reducing all-cause mortality as well as ischemic and bleeding complications.

Whereas the results of some studies chime in with the results of the present study, there are studies that have suggested further investigation to determine the optimal strategy for heparin administration.^4^^,^^6^ Miller^6^ investigated the current patterns of the use of heparin in angiography and suggested that further studies be done on the administration of heparin as an anticoagulant. 

Some studies have reported increased risk of hematoma post administration of heparin. A study which was conducted on 322 patients to assess hematoma and its risk factors reported that the use of anticoagulant agents might increase the risk of the occurrence of hematoma.^17^ On the other hand, previous case reports have shown the increased risk of pituitary apoplexy and perirenal hematoma following coronary angiography in patients who had used anticoagulant agents.^18^^,^^19^

According to some textbooks, there is no absolute indication for administering routine intravenous heparin during coronary angiography through the femoral approach. However, in the case of patients at high risk of thromboembolic complications (for example, in conditions such as severe aortic stenosis, severe peripheral vascular disease, and long use of the guide wire in the peripheral blood flow), heparin administration is advised. Absolute indication exists in the radial and brachial approaches.^20^ Other textbooks have generally suggested the intravenous administration of 2-3 thousands units of heparin upon catheterization, without defining any indications.^2^

An important limitation of this study was the fact that we did not record the exact duration of the procedure. Moreover, we merely excluded procedures which lasted more than 30 minutes. Not only does this point contrast with other studies in which timing was more accurately measured, but also this point was important in the prevalence of complications. Nonetheless, angiography is a diagnostic procedure that, aside from the skill of the operator, is strongly dependent on the anatomical status and tortuosity of the abdominal and thoracic aortas. This may lead to catheter and guide-wire exchange, necessitate the use of various sizes, and as such increase the risk of the complications. It is also deserving of note, that in some cases, we had no choice but to administer heparin because of the prolonged procedure time. 

## Conclusion

Administration of heparin during coronary artery angiography had no significant effect on the occurrence of hemorrhagic, ischemic, and vascular complications in our study population. Our findings suggest that when risk factors for thromboembolism are low, coronary angiography could be safely performed without the administration of heparin through the femoral route. In addition, local complications were not increased by the use of heparin in our patients.
